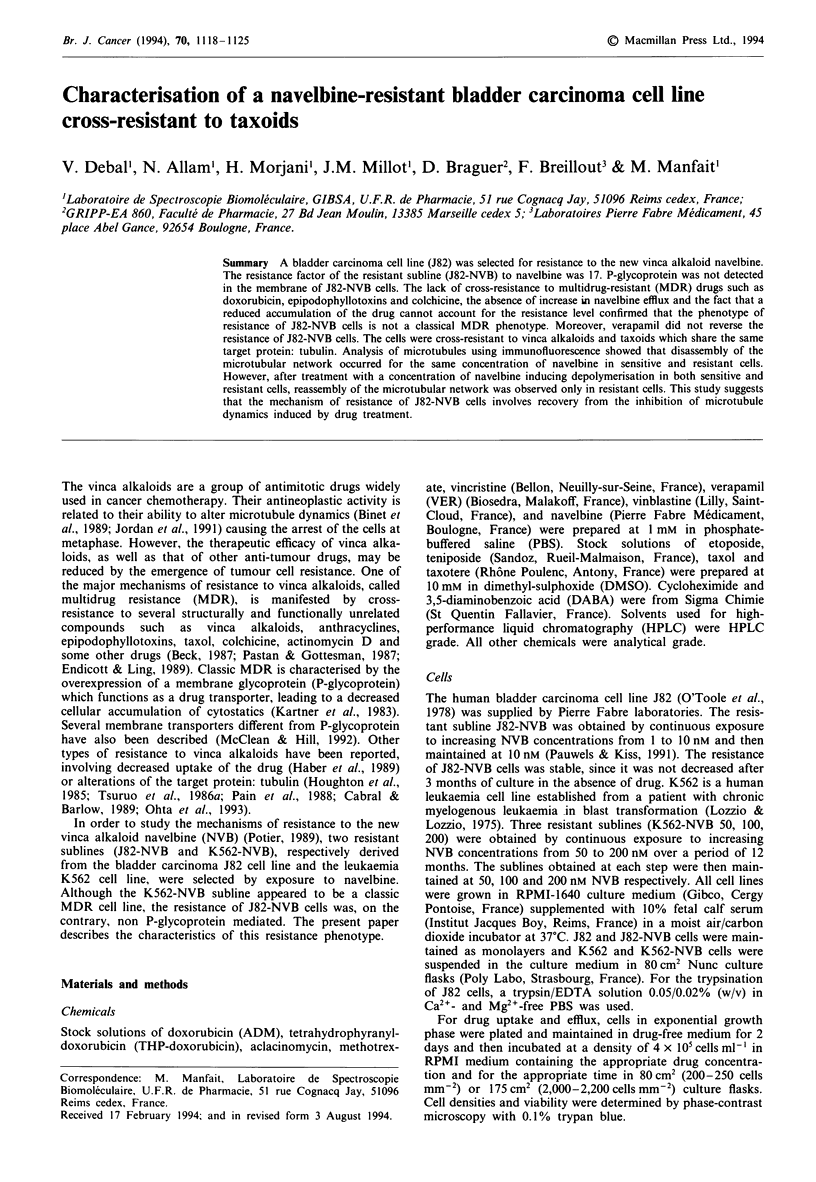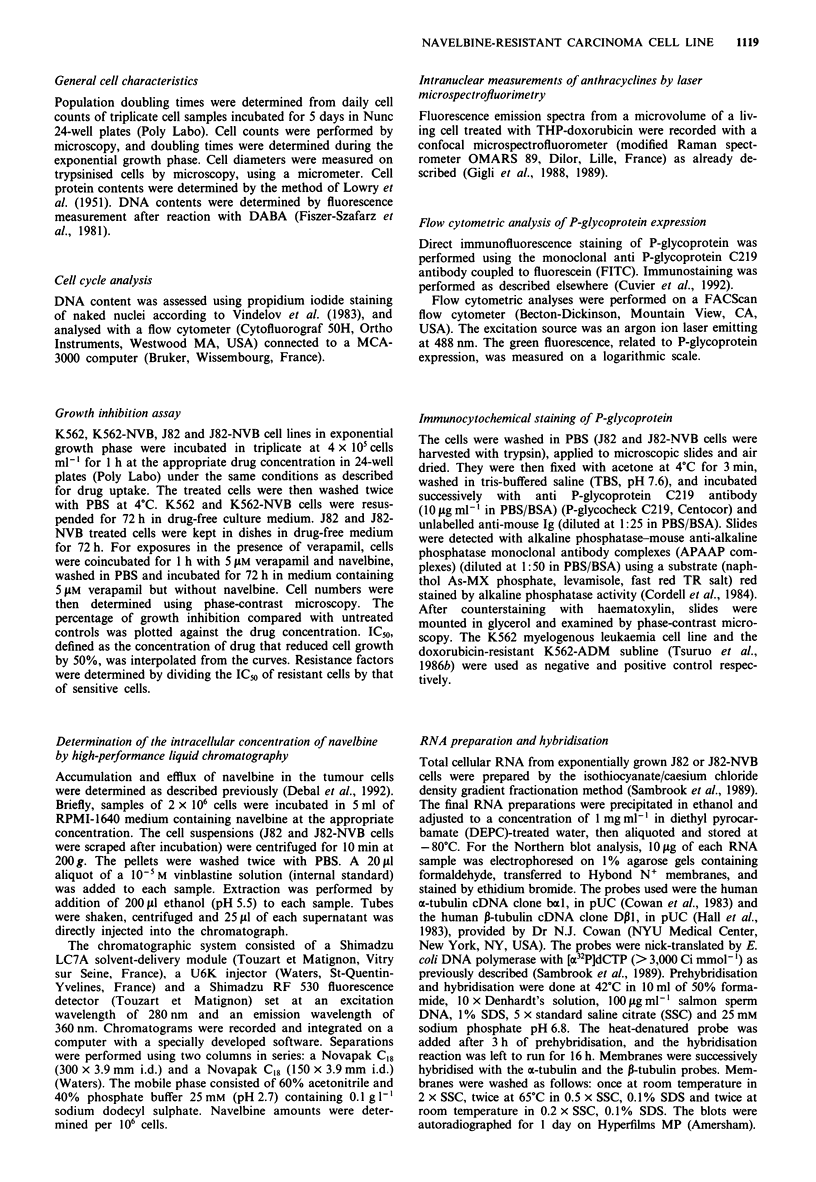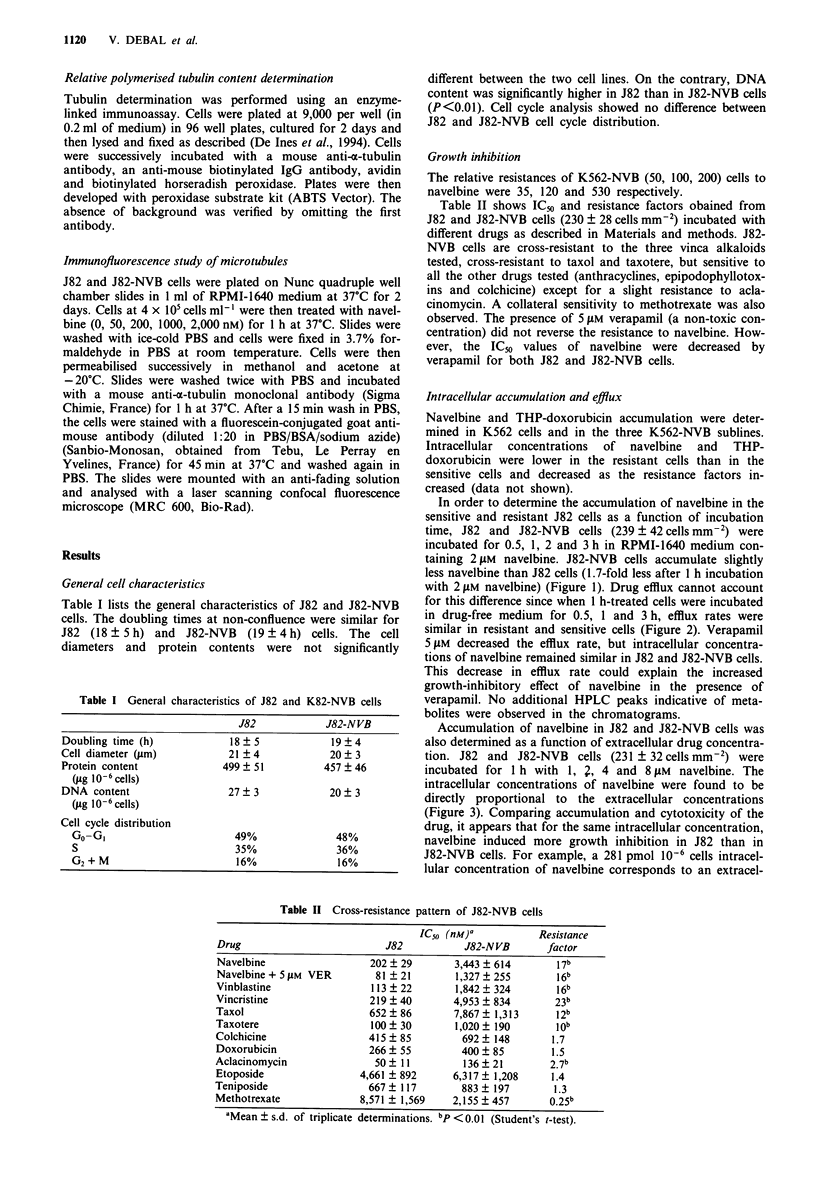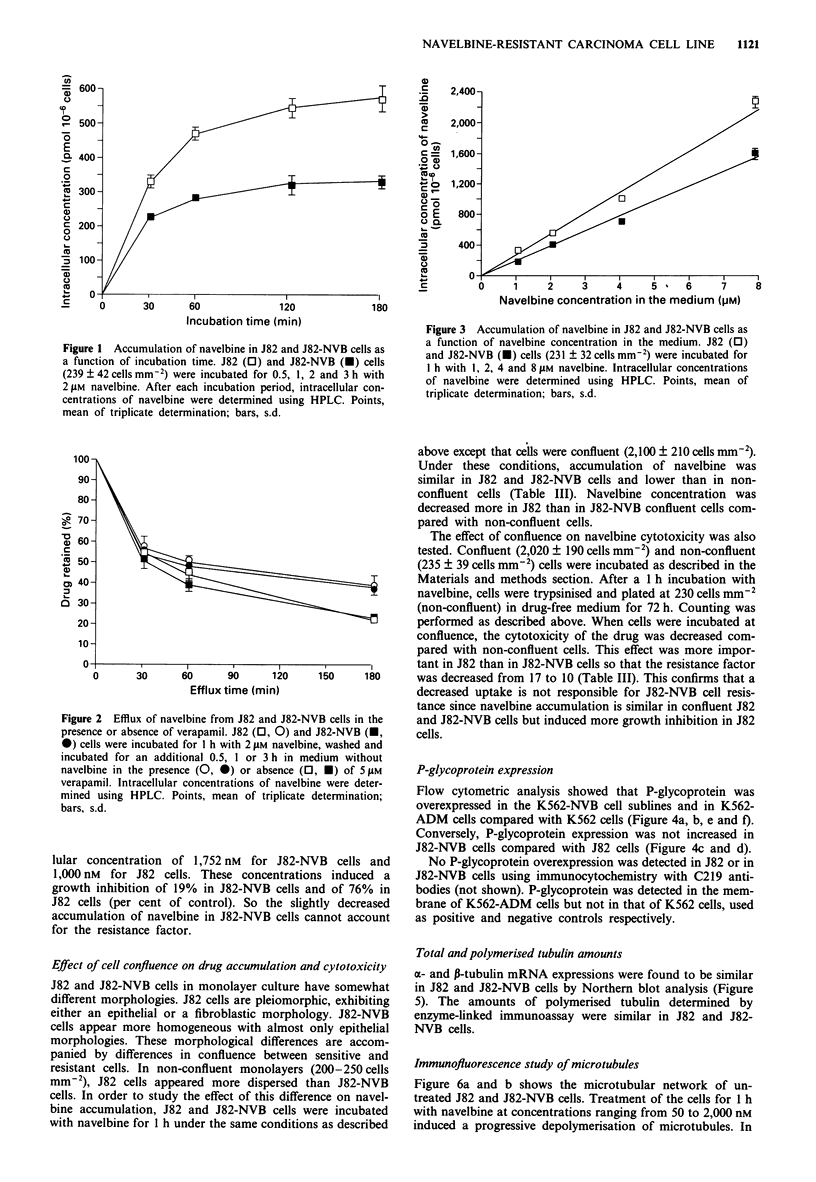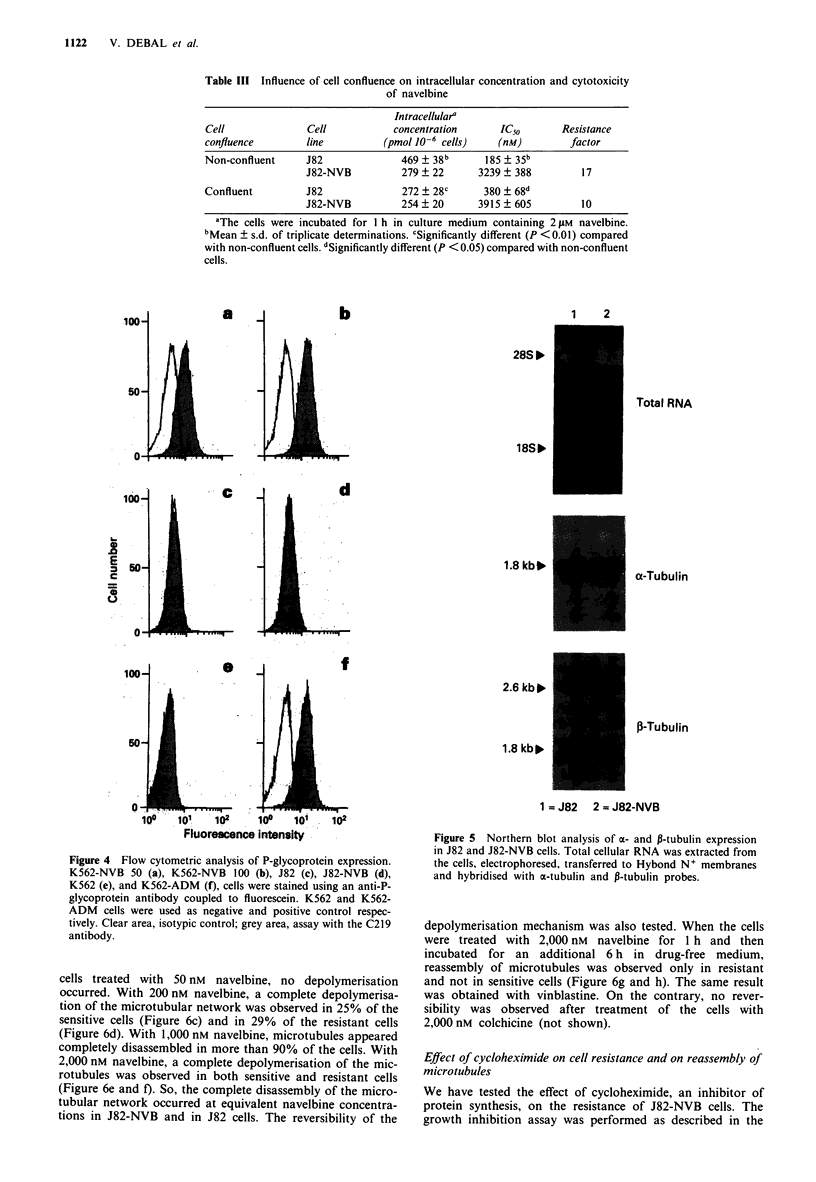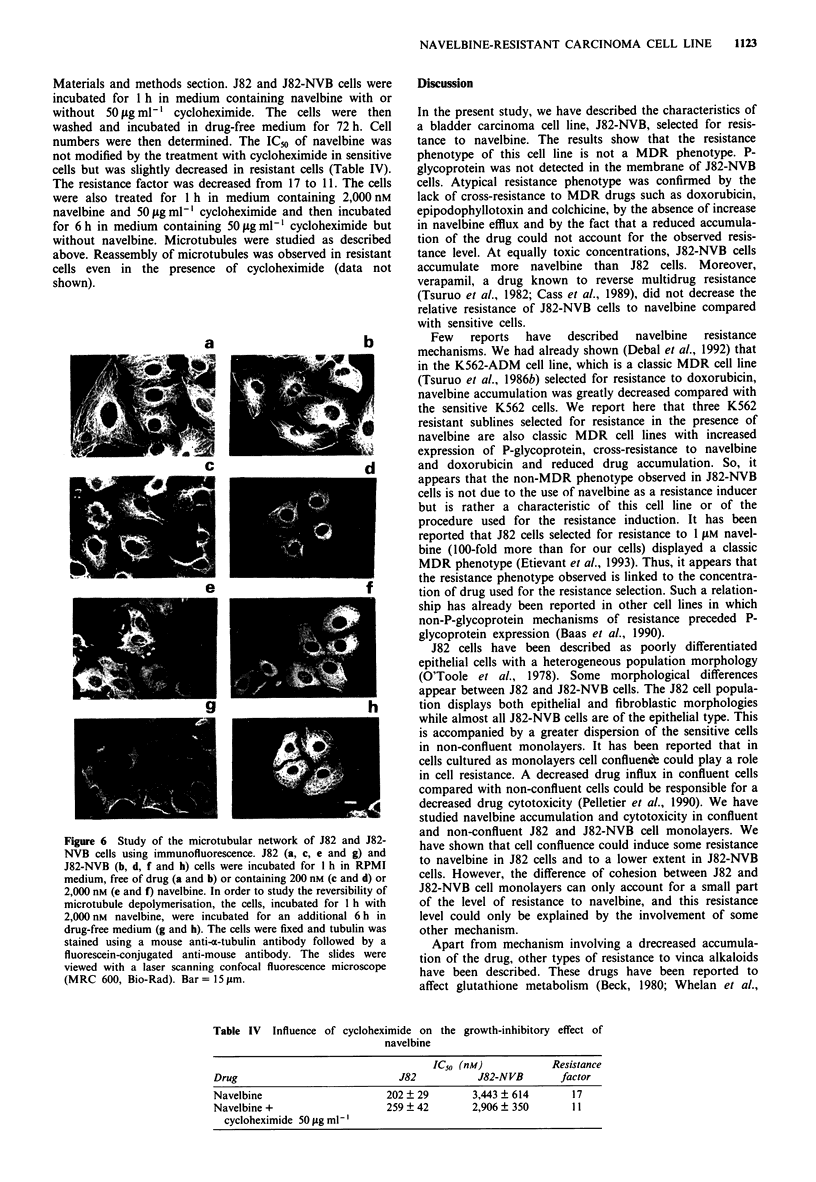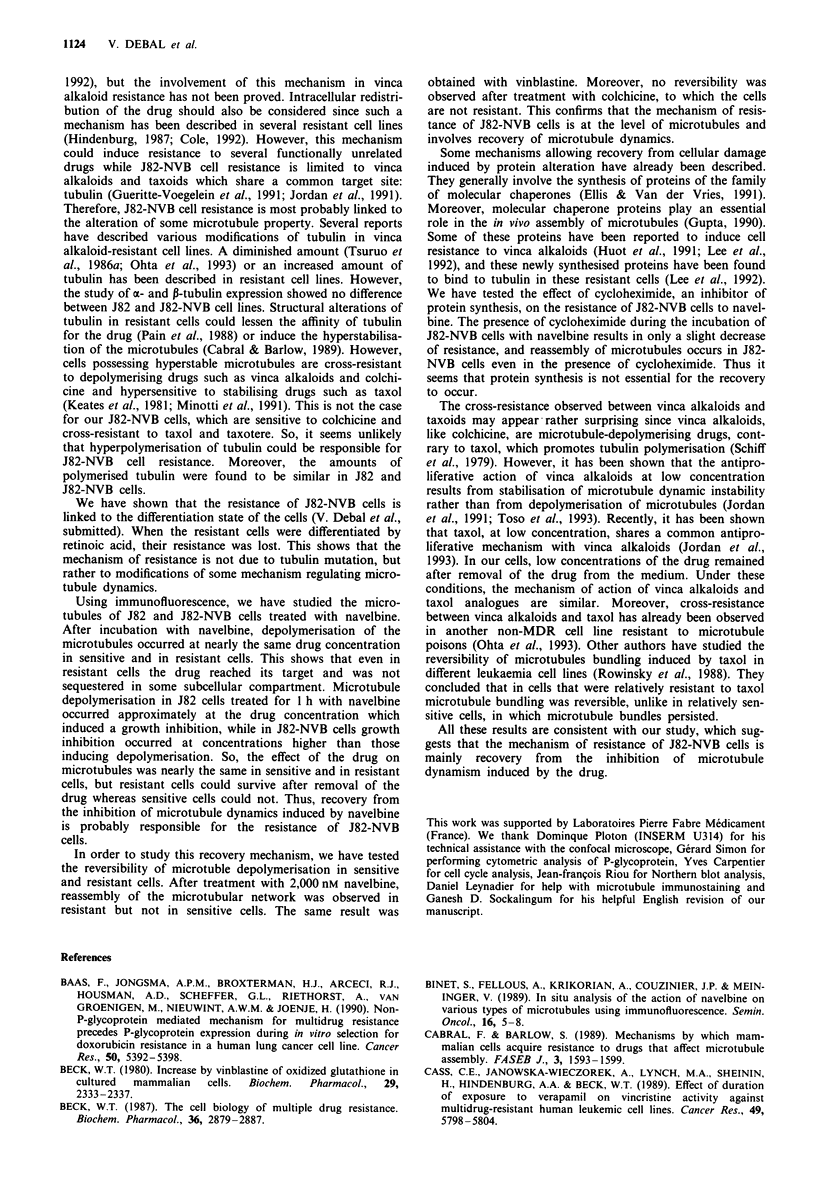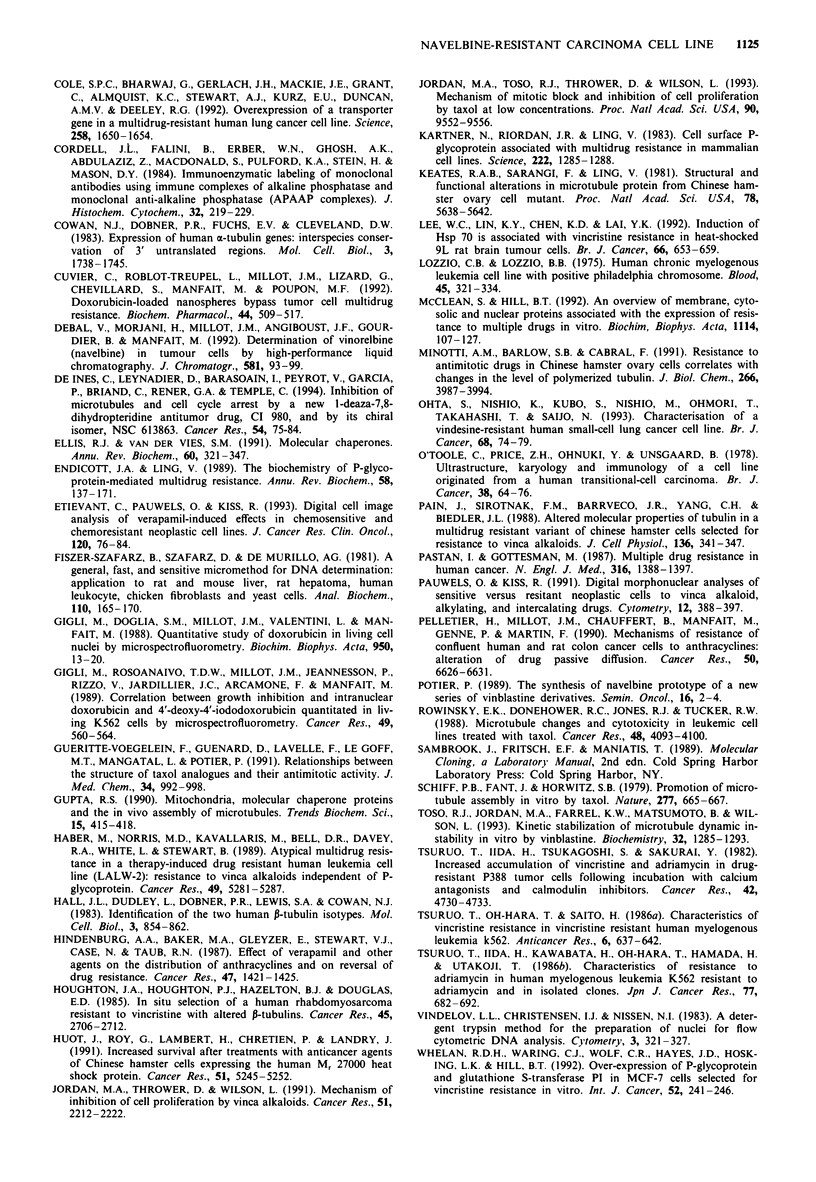# Characterisation of a navelbine-resistant bladder carcinoma cell line cross-resistant to taxoids.

**DOI:** 10.1038/bjc.1994.458

**Published:** 1994-12

**Authors:** V. Debal, N. Allam, H. Morjani, J. M. Millot, D. Braguer, F. Breillout, M. Manfait

**Affiliations:** Laboratoire de Spectroscopie Biomoléculaire, GIBSA, U.F.R. de Pharmacie, Reims, France.

## Abstract

**Images:**


Br. J. Cancer (1994), 70, 1118–1125 © Macmillan Press Ltd., 1994

### Characterisation of a navelbine-resistant bladder carcinoma cell line cross-resistant to taxoids

V. Debal[1], N. Allam[1], H. Morjani[1], J.M. Millot[1], D. Braguer[2], F. Breillout[3] & M. Manfait[1]

[1]*Laboratoire de Spectroscopie Biomoléculaire, GIBSA, U.F.R. de Pharmacie, 51 rue Cognacq Jay, 51096 Reims cedex, France;* [2]*GRIPP-EA 860, Faculté de Pharmacie, 27 Bd Jean Moulin, 13385 Marseille cedex 5;* [3]*Laboratoires Pierre Fabre Médicament, 45 place Abel Gance, 92654 Boulogne, France.*

**Summary** A bladder carcinoma cell line (J82) was selected for resistance to the new vinca alkaloid navelbine. The resistance factor of the resistant subline (J82-NVB) to navelbine was 17. P-glycoprotein was not detected in the membrane of J82-NVB cells. The lack of cross-resistance to multidrug-resistant (MDR) drugs such as doxorubicin, epipodophyllotoxins and colchicine, the absence of increase in navelbine efflux and the fact that a reduced accumulation of the drug cannot account for the resistance level confirmed that the phenotype of resistance of J82-NVB cells is not a classical MDR phenotype. Moreover, verapamil did not reverse the resistance of J82-NVB cells. The cells were cross-resistant to vinca alkaloids and taxoids which share the same target protein: tubulin. Analysis of microtubules using immunofluorescence showed that disassembly of the microtubular network occurred for the same concentration of navelbine in sensitive and resistant cells. However, after treatment with a concentration of navelbine inducing depolymerisation in both sensitive and resistant cells, reassembly of the microtubular network was observed only in resistant cells. This study suggests that the mechanism of resistance of J82-NVB cells involves recovery from the inhibition of microtubule dynamics induced by drug treatment.

The vinca alkaloids are a group of antimitotic drugs widely used in cancer chemotherapy. Their antineoplastic activity is related to their ability to alter microtubule dynamics (Binet *et al.*, 1989; Jordan *et al.*, 1991) causing the arrest of the cells at metaphase. However, the therapeutic efficacy of vinca alkaloids, as well as that of other anti-tumour drugs, may be reduced by the emergence of tumour cell resistance. One of the major mechanisms of resistance to vinca alkaloids, called multidrug resistance (MDR), is manifested by cross-resistance to several structurally and functionally unrelated compounds such as vinca alkaloids, anthracyclines, epipodophyllotoxins, taxol, colchicine, actinomycin D and some other drugs (Beck, 1987; Pastan & Gottesman, 1987; Endicott & Ling, 1989). Classic MDR is characterised by the overexpression of a membrane glycoprotein (P-glycoprotein) which functions as a drug transporter, leading to a decreased cellular accumulation of cytostatics (Kartner *et al.*, 1983). Several membrane transporters different from P-glycoprotein have also been described (McClean & Hill, 1992). Other types of resistance to vinca alkaloids have been reported, involving decreased uptake of the drug (Haber *et al.*, 1989) or alterations of the target protein: tubulin (Houghton *et al.*, 1985; Tsuruo *et al.*, 1986*a*; Pain *et al.*, 1988; Cabral & Barlow, 1989; Ohta *et al.*, 1993).

In order to study the mechanisms of resistance to the new vinca alkaloid navelbine (NVB) (Potier, 1989), two resistant sublines (J82-NVB and K562-NVB), respectively derived from the bladder carcinoma J82 cell line and the leukaemia K562 cell line, were selected by exposure to navelbine. Although the K562-NVB subline appeared to be a classic MDR cell line, the resistance of J82-NVB cells was, on the contrary, non P-glycoprotein mediated. The present paper describes the characteristics of this resistance phenotype.

### Materials and methods

### *Chemicals*

Stock solutions of doxorubicin (ADM), tetrahydrophyranyl-doxorubicin (THP-doxorubicin), aclacinomycin, methotrexate, vincristine (Bellon, Neuilly-sur-Seine, France), verapamil (VER) (Biosedra, Malakoff, France), vinblastine (Lilly, Saint-Cloud, France), and navelbine (Pierre Fabre Médicament, Boulogne, France) were prepared at 1 mM in phosphate-buffered saline (PBS). Stock solutions of etoposide, teniposide (Sandoz, Rueil-Malmaison, France), taxol and taxotere (Rhône Poulenc, Antony, France) were prepared at 10 mM in dimethyl-sulphoxide (DMSO). Cycloheximide and 3,5-diaminobenzoic acid (DABA) were from Sigma Chimie (St Quentin Fallavier, France). Solvents used for high-performance liquid chromatography (HPLC) were HPLC grade. All other chemicals were analytical grade.

### *Cells*

The human bladder carcinoma cell line J82 (O'Toole *et al.*, 1978) was supplied by Pierre Fabre laboratories. The resistant subline J82-NVB was obtained by continuous exposure to increasing NVB concentrations from 1 to 10 nM and then maintained at 10 nM (Pauwels & Kiss, 1991). The resistance of J82-NVB cells was stable, since it was not decreased after 3 months of culture in the absence of drug. K562 is a human leukaemia cell line established from a patient with chronic myelogenous leukaemia in blast transformation (Lozzio & Lozzio, 1975). Three resistant sublines (K562-NVB 50, 100, 200) were obtained by continuous exposure to increasing NVB concentrations from 50 to 200 nM over a period of 12 months. The sublines obtained at each step were then maintained at 50, 100 and 200 nM NVB respectively. All cell lines were grown in RPMI-1640 culture medium (Gibco, Cergy Pontoise, France) supplemented with 10% fetal calf serum (Institut Jacques Boy, Reims, France) in a moist air/carbon dioxide incubator at 37°C. J82 and J82-NVB cells were maintained as monolayers and K562 and K562-NVB cells were suspended in the culture medium in 80 $cm^2$ Nunc culture flasks (Poly Labo, Strasbourg, France). For the trypsination of J82 cells, a trypsin/EDTA solution 0.05/0.02% (w/v) in $Ca^{2+}$- and $Mg^{2+}$-free PBS was used.

For drug uptake and efflux, cells in exponential growth phase were plated and maintained in drug-free medium for 2 days and then incubated at a density of $4 \times 10^5$ cells $ml^{-1}$ in RPMI medium containing the appropriate drug concentration and for the appropriate time in 80 $cm^2$ (200–250 cells $mm^{-2}$) or 175 $cm^2$ (2,000–2,200 cells $mm^{-2}$) culture flasks. Cell densities and viability were determined by phase-contrast microscopy with 0.1% trypan blue.

Correspondence: M. Manfait, Laboratoire de Spectroscopie Biomoléculaire, U.F.R. de Pharmacie, 51 rue Cognacq Jay, 51096 Reims cedex, France.
Received 17 February 1994; and in revised form 3 August 1994.

*General cell characteristics*

Population doubling times were determined from daily cell counts of triplicate cell samples incubated for 5 days in Nunc 24-well plates (Poly Labo). Cell counts were performed by microscopy, and doubling times were determined during the exponential growth phase. Cell diameters were measured on trypsinised cells by microscopy, using a micrometer. Cell protein contents were determined by the method of Lowry *et al.* (1951). DNA contents were determined by fluorescence measurement after reaction with DABA (Fiszer-Szafarz *et al.*, 1981).

*Cell cycle analysis*

DNA content was assessed using propidium iodide staining of naked nuclei according to Vindelov *et al.* (1983), and analysed with a flow cytometer (Cytofluorograf 50H, Ortho Instruments, Westwood MA, USA) connected to a MCA-3000 computer (Bruker, Wissembourg, France).

*Growth inhibition assay*

K562, K562-NVB, J82 and J82-NVB cell lines in exponential growth phase were incubated in triplicate at $4 \times 10^5$ cells $ml^{-1}$ for 1 h at the appropriate drug concentration in 24-well plates (Poly Labo) under the same conditions as described for drug uptake. The treated cells were then washed twice with PBS at 4°C. K562 and K562-NVB cells were resuspended for 72 h in drug-free culture medium. J82 and J82-NVB treated cells were kept in dishes in drug-free medium for 72 h. For exposures in the presence of verapamil, cells were coincubated for 1 h with 5 μM verapamil and navelbine, washed in PBS and incubated for 72 h in medium containing 5 μM verapamil but without navelbine. Cell numbers were then determined using phase-contrast microscopy. The percentage of growth inhibition compared with untreated controls was plotted against the drug concentration. $IC_{50}$, defined as the concentration of drug that reduced cell growth by 50%, was interpolated from the curves. Resistance factors were determined by dividing the $IC_{50}$ of resistant cells by that of sensitive cells.

*Determination of the intracellular concentration of navelbine by high-performance liquid chromatography*

Accumulation and efflux of navelbine in the tumour cells were determined as described previously (Debal *et al.*, 1992). Briefly, samples of $2 \times 10^6$ cells were incubated in 5 ml of RPMI-1640 medium containing navelbine at the appropriate concentration. The cell suspensions (J82 and J82-NVB cells were scraped after incubation) were centrifuged for 10 min at 200 *g*. The pellets were washed twice with PBS. A 20 μl aliquot of a $10^{-5}$ M vinblastine solution (internal standard) was added to each sample. Extraction was performed by addition of 200 μl ethanol (pH 5.5) to each sample. Tubes were shaken, centrifuged and 25 μl of each supernatant was directly injected into the chromatograph.

The chromatographic system consisted of a Shimadzu LC7A solvent-delivery module (Touzart et Matignon, Vitry sur Seine, France), a U6K injector (Waters, St-Quentin-Yvelines, France) and a Shimadzu RF 530 fluorescence detector (Touzart et Matignon) set at an excitation wavelength of 280 nm and an emission wavelength of 360 nm. Chromatograms were recorded and integrated on a computer with a specially developed software. Separations were performed using two columns in series: a Novapak $C_{18}$ (300 × 3.9 mm i.d.) and a Novapak $C_{18}$ (150 × 3.9 mm i.d.) (Waters). The mobile phase consisted of 60% acetonitrile and 40% phosphate buffer 25 mM (pH 2.7) containing 0.1 g $l^{-1}$ sodium dodecyl sulphate. Navelbine amounts were determined per $10^6$ cells.

*Intranuclear measurements of anthracyclines by laser microspectrofluorimetry*

Fluorescence emission spectra from a microvolume of a living cell treated with THP-doxorubicin were recorded with a confocal microspectrofluorometer (modified Raman spectrometer OMARS 89, Dilor, Lille, France) as already described (Gigli *et al.*, 1988, 1989).

*Flow cytometric analysis of P-glycoprotein expression*

Direct immunofluorescence staining of P-glycoprotein was performed using the monoclonal anti P-glycoprotein C219 antibody coupled to fluorescein (FITC). Immunostaining was performed as described elsewhere (Cuvier *et al.*, 1992).

Flow cytometric analyses were performed on a FACScan flow cytometer (Becton-Dickinson, Mountain View, CA, USA). The excitation source was an argon ion laser emitting at 488 nm. The green fluorescence, related to P-glycoprotein expression, was measured on a logarithmic scale.

*Immunocytochemical staining of P-glycoprotein*

The cells were washed in PBS (J82 and J82-NVB cells were harvested with trypsin), applied to microscopic slides and air dried. They were then fixed with acetone at 4°C for 3 min, washed in tris-buffered saline (TBS, pH 7.6), and incubated successively with anti P-glycoprotein C219 antibody (10 μg $ml^{-1}$ in PBS/BSA) (P-glycocheck C219, Centocor) and unlabelled anti-mouse Ig (diluted at 1:25 in PBS/BSA). Slides were detected with alkaline phosphatase–mouse anti-alkaline phosphatase monoclonal antibody complexes (APAAP complexes) (diluted at 1:50 in PBS/BSA) using a substrate (naphthol As-MX phosphate, levamisole, fast red TR salt) red stained by alkaline phosphatase activity (Cordell *et al.*, 1984). After counterstaining with haematoxylin, slides were mounted in glycerol and examined by phase-contrast microscopy. The K562 myelogenous leukaemia cell line and the doxorubicin-resistant K562-ADM subline (Tsuruo *et al.*, 1986*b*) were used as negative and positive control respectively.

*RNA preparation and hybridisation*

Total cellular RNA from exponentially grown J82 or J82-NVB cells were prepared by the isothiocyanate/caesium chloride density gradient fractionation method (Sambrook *et al.*, 1989). The final RNA preparations were precipitated in ethanol and adjusted to a concentration of 1 mg $ml^{-1}$ in diethyl pyrocarbamate (DEPC)-treated water, then aliquoted and stored at −80°C. For the Northern blot analysis, 10 μg of each RNA sample was electrophoresed on 1% agarose gels containing formaldehyde, transferred to Hybond $N^+$ membranes, and stained by ethidium bromide. The probes used were the human α-tubulin cDNA clone bα1, in pUC (Cowan *et al.*, 1983) and the human β-tubulin cDNA clone Dβ1, in pUC (Hall *et al.*, 1983), provided by Dr N.J. Cowan (NYU Medical Center, New York, NY, USA). The probes were nick-translated by *E. coli* DNA polymerase with [$\alpha^{32}$P]dCTP (> 3,000 Ci $mmol^{-1}$) as previously described (Sambrook *et al.*, 1989). Prehybridisation and hybridisation were done at 42°C in 10 ml of 50% formamide, 10 × Denhardt's solution, 100 μg $ml^{-1}$ salmon sperm DNA, 1% SDS, 5 × standard saline citrate (SSC) and 25 mM sodium phosphate pH 6.8. The heat-denatured probe was added after 3 h of prehybridisation, and the hybridisation reaction was left to run for 16 h. Membranes were successively hybridised with the α-tubulin and the β-tubulin probes. Membranes were washed as follows: once at room temperature in 2 × SSC, twice at 65°C in 0.5 × SSC, 0.1% SDS and twice at room temperature in 0.2 × SSC, 0.1% SDS. The blots were autoradiographed for 1 day on Hyperfilms MP (Amersham).

*Relative polymerised tubulin content determination*

Tubulin determination was performed using an enzyme-linked immunoassay. Cells were plated at 9,000 per well (in 0.2 ml of medium) in 96 well plates, cultured for 2 days and then lysed and fixed as described (De Ines *et al*., 1994). Cells were successively incubated with a mouse anti-α-tubulin antibody, an anti-mouse biotinylated IgG antibody, avidin and biotinylated horseradish peroxidase. Plates were then developed with peroxidase substrate kit (ABTS Vector). The absence of background was verified by omitting the first antibody.

*Immunofluorescence study of microtubules*

J82 and J82-NVB cells were plated on Nunc quadruple well chamber slides in 1 ml of RPMI-1640 medium at 37°C for 2 days. Cells at $4 \times 10^5$ cells $ml^{-1}$ were then treated with navelbine (0, 50, 200, 1000, 2,000 nM) for 1 h at 37°C. Slides were washed with ice-cold PBS and cells were fixed in 3.7% formaldehyde in PBS at room temperature. Cells were then permeabilised successively in methanol and acetone at − 20°C. Slides were washed twice with PBS and incubated with a mouse anti-α-tubulin monoclonal antibody (Sigma Chimie, France) for 1 h at 37°C. After a 15 min wash in PBS, the cells were stained with a fluorescein-conjugated goat anti-mouse antibody (diluted 1:20 in PBS/BSA/sodium azide) (Sanbio-Monosan, obtained from Tebu, Le Perray en Yvelines, France) for 45 min at 37°C and washed again in PBS. The slides were mounted with an anti-fading solution and analysed with a laser scanning confocal fluorescence microscope (MRC 600, Bio-Rad).

### Results

*General cell characteristics*

Table I lists the general characteristics of J82 and J82-NVB cells. The doubling times at non-confluence were similar for J82 (18 ± 5 h) and J82-NVB (19 ± 4 h) cells. The cell diameters and protein contents were not significantly different between the two cell lines. On the contrary, DNA content was significantly higher in J82 than in J82-NVB cells ($P<0.01$). Cell cycle analysis showed no difference between J82 and J82-NVB cell cycle distribution.

**Table I** General characteristics of J82 and K82-NVB cells

| | *J82* | *J82-NVB* |
|---|---|---|
| Doubling time (h) | 18 ± 5 | 19 ± 4 |
| Cell diameter (μm) | 21 ± 4 | 20 ± 3 |
| Protein content (μg $10^{-6}$ cells) | 499 ± 51 | 457 ± 46 |
| DNA content (μg $10^{-6}$ cells) | 27 ± 3 | 20 ± 3 |
| Cell cycle distribution | | |
| $G_0$–$G_1$ | 49% | 48% |
| S | 35% | 36% |
| $G_2$ + M | 16% | 16% |

*Growth inhibition*

The relative resistances of K562-NVB (50, 100, 200) cells to navelbine were 35, 120 and 530 respectively.

Table II shows $IC_{50}$ and resistance factors obained from J82 and J82-NVB cells (230 ± 28 cells $mm^{-2}$) incubated with different drugs as described in Materials and methods. J82-NVB cells are cross-resistant to the three vinca alkaloids tested, cross-resistant to taxol and taxotere, but sensitive to all the other drugs tested (anthracyclines, epipodophyllotoxins and colchicine) except for a slight resistance to aclacinomycin. A collateral sensitivity to methotrexate was also observed. The presence of 5 μM verapamil (a non-toxic concentration) did not reverse the resistance to navelbine. However, the $IC_{50}$ values of navelbine were decreased by verapamil for both J82 and J82-NVB cells.

*Intracellular accumulation and efflux*

Navelbine and THP-doxorubicin accumulation were determined in K562 cells and in the three K562-NVB sublines. Intracellular concentrations of navelbine and THP-doxorubicin were lower in the resistant cells than in the sensitive cells and decreased as the resistance factors increased (data not shown).

In order to determine the accumulation of navelbine in the sensitive and resistant J82 cells as a function of incubation time, J82 and J82-NVB cells (239 ± 42 cells $mm^{-2}$) were incubated for 0.5, 1, 2 and 3 h in RPMI-1640 medium containing 2 μM navelbine. J82-NVB cells accumulate slightly less navelbine than J82 cells (1.7-fold less after 1 h incubation with 2 μM navelbine) (Figure 1). Drug efflux cannot account for this difference since when 1 h-treated cells were incubated in drug-free medium for 0.5, 1 and 3 h, efflux rates were similar in resistant and sensitive cells (Figure 2). Verapamil 5 μM decreased the efflux rate, but intracellular concentrations of navelbine remained similar in J82 and J82-NVB cells. This decrease in efflux rate could explain the increased growth-inhibitory effect of navelbine in the presence of verapamil. No additional HPLC peaks indicative of metabolites were observed in the chromatograms.

Accumulation of navelbine in J82 and J82-NVB cells was also determined as a function of extracellular drug concentration. J82 and J82-NVB cells (231 ± 32 cells $mm^{-2}$) were incubated for 1 h with 1, 2, 4 and 8 μM navelbine. The intracellular concentrations of navelbine were found to be directly proportional to the extracellular concentrations (Figure 3). Comparing accumulation and cytotoxicity of the drug, it appears that for the same intracellular concentration, navelbine induced more growth inhibition in J82 than in J82-NVB cells. For example, a 281 pmol $10^{-6}$ cells intracellular concentration of navelbine corresponds to an extracel-

**Table II** Cross-resistance pattern of J82-NVB cells

| *Drug* | *$IC_{50}$ (nM)[a]* *J82* | *J82-NVB* | *Resistance factor* |
|---|---|---|---|
| Navelbine | 202 ± 29 | 3,443 ± 614 | 17[b] |
| Navelbine + 5 μM VER | 81 ± 21 | 1,327 ± 255 | 16[b] |
| Vinblastine | 113 ± 22 | 1,842 ± 324 | 16[b] |
| Vincristine | 219 ± 40 | 4,953 ± 834 | 23[b] |
| Taxol | 652 ± 86 | 7,867 ± 1,313 | 12[b] |
| Taxotere | 100 ± 30 | 1,020 ± 190 | 10[b] |
| Colchicine | 415 ± 85 | 692 ± 148 | 1.7 |
| Doxorubicin | 266 ± 55 | 400 ± 85 | 1.5 |
| Aclacinomycin | 50 ± 11 | 136 ± 21 | 2.7[b] |
| Etoposide | 4,661 ± 892 | 6,317 ± 1,208 | 1.4 |
| Teniposide | 667 ± 117 | 883 ± 197 | 1.3 |
| Methotrexate | 8,571 ± 1,569 | 2,155 ± 457 | 0.25[b] |

[a]Mean ± s.d. of triplicate determinations. [b]$P<0.01$ (Student's *t*-test).

**Figure 1** Accumulation of navelbine in J82 and J82-NVB cells as a function of incubation time. J82 (□) and J82-NVB (■) cells (239 ± 42 cells $mm^{-2}$) were incubated for 0.5, 1, 2 and 3 h with 2 μM navelbine. After each incubation period, intracellular concentrations of navelbine were determined using HPLC. Points, mean of triplicate determination; bars, s.d.

**Figure 2** Efflux of navelbine from J82 and J82-NVB cells in the presence or absence of verapamil. J82 (□, ○) and J82-NVB (■, ●) cells were incubated for 1 h with 2 μM navelbine, washed and incubated for an additional 0.5, 1 or 3 h in medium without navelbine in the presence (○, ●) or absence (□, ■) of 5 μM verapamil. Intracellular concentrations of navelbine were determined using HPLC. Points, mean of triplicate determination; bars, s.d.

lular concentration of 1,752 nM for J82-NVB cells and 1,000 nM for J82 cells. These concentrations induced a growth inhibition of 19% in J82-NVB cells and of 76% in J82 cells (per cent of control). So the slightly decreased accumulation of navelbine in J82-NVB cells cannot account for the resistance factor.

*Effect of cell confluence on drug accumulation and cytotoxicity*

J82 and J82-NVB cells in monolayer culture have somewhat different morphologies. J82 cells are pleiomorphic, exhibiting either an epithelial or a fibroblastic morphology. J82-NVB cells appear more homogeneous with almost only epithelial morphologies. These morphological differences are accompanied by differences in confluence between sensitive and resistant cells. In non-confluent monolayers (200–250 cells $mm^{-2}$), J82 cells appeared more dispersed than J82-NVB cells. In order to study the effect of this difference on navelbine accumulation, J82 and J82-NVB cells were incubated with navelbine for 1 h under the same conditions as described

**Figure 3** Accumulation of navelbine in J82 and J82-NVB cells as a function of navelbine concentration in the medium. J82 (□) and J82-NVB (■) cells (231 ± 32 cells $mm^{-2}$) were incubated for 1 h with 1, 2, 4 and 8 μM navelbine. Intracellular concentrations of navelbine were determined using HPLC. Points, mean of triplicate determination; bars, s.d.

above except that cells were confluent (2,100 ± 210 cells $mm^{-2}$). Under these conditions, accumulation of navelbine was similar in J82 and J82-NVB cells and lower than in non-confluent cells (Table III). Navelbine concentration was decreased more in J82 than in J82-NVB confluent cells compared with non-confluent cells.

The effect of confluence on navelbine cytotoxicity was also tested. Confluent (2,020 ± 190 cells $mm^{-2}$) and non-confluent (235 ± 39 cells $mm^{-2}$) cells were incubated as described in the Materials and methods section. After a 1 h incubation with navelbine, cells were trypsinised and plated at 230 cells $mm^{-2}$ (non-confluent) in drug-free medium for 72 h. Counting was performed as described above. When cells were incubated at confluence, the cytotoxicity of the drug was decreased compared with non-confluent cells. This effect was more important in J82 than in J82-NVB cells so that the resistance factor was decreased from 17 to 10 (Table III). This confirms that a decreased uptake is not responsible for J82-NVB cell resistance since navelbine accumulation is similar in confluent J82 and J82-NVB cells but induced more growth inhibition in J82 cells.

*P-glycoprotein expression*

Flow cytometric analysis showed that P-glycoprotein was overexpressed in the K562-NVB cell sublines and in K562-ADM cells compared with K562 cells (Figure 4a, b, e and f). Conversely, P-glycoprotein expression was not increased in J82-NVB cells compared with J82 cells (Figure 4c and d).

No P-glycoprotein overexpression was detected in J82 or in J82-NVB cells using immunocytochemistry with C219 antibodies (not shown). P-glycoprotein was detected in the membrane of K562-ADM cells but not in that of K562 cells, used as positive and negative controls respectively.

*Total and polymerised tubulin amounts*

α- and β-tubulin mRNA expressions were found to be similar in J82 and J82-NVB cells by Northern blot analysis (Figure 5). The amounts of polymerised tubulin determined by enzyme-linked immunoassay were similar in J82 and J82-NVB cells.

*Immunofluorescence study of microtubules*

Figure 6a and b shows the microtubular network of untreated J82 and J82-NVB cells. Treatment of the cells for 1 h with navelbine at concentrations ranging from 50 to 2,000 nM induced a progressive depolymerisation of microtubules. In

**Table III** Influence of cell confluence on intracellular concentration and cytotoxicity of navelbine

| *Cell confluence* | *Cell line* | *Intracellular[a] concentration (pmol $10^{-6}$ cells)* | *$IC_{50}$ (nM)* | *Resistance factor* |
|---|---|---|---|---|
| Non-confluent | J82 | 469 ± 38[b] | 185 ± 35[b] | |
| | J82-NVB | 279 ± 22 | 3239 ± 388 | 17 |
| Confluent | J82 | 272 ± 28[c] | 380 ± 68[d] | |
| | J82-NVB | 254 ± 20 | 3915 ± 605 | 10 |

[a]The cells were incubated for 1 h in culture medium containing 2 μM navelbine. [b]Mean ± s.d. of triplicate determinations. [c]Significantly different ($P < 0.01$) compared with non-confluent cells. [d]Significantly different ($P < 0.05$) compared with non-confluent cells.

**Figure 4** Flow cytometric analysis of P-glycoprotein expression. K562-NVB 50 (**a**), K562-NVB 100 (**b**), J82 (**c**), J82-NVB (**d**), K562 (**e**), and K562-ADM (**f**), cells were stained using an anti-P-glycoprotein antibody coupled to fluorescein. K562 and K562-ADM cells were used as negative and positive control respectively. Clear area, isotypic control; grey area, assay with the C219 antibody.

**Figure 5** Northern blot analysis of α- and β-tubulin expression in J82 and J82-NVB cells. Total cellular RNA was extracted from the cells, electrophoresed, transferred to Hybond N+ membranes and hybridised with α-tubulin and β-tubulin probes.

cells treated with 50 nM navelbine, no depolymerisation occurred. With 200 nM navelbine, a complete depolymerisation of the microtubular network was observed in 25% of the sensitive cells (Figure 6c) and in 29% of the resistant cells (Figure 6d). With 1,000 nM navelbine, microtubules appeared completely disassembled in more than 90% of the cells. With 2,000 nM navelbine, a complete depolymerisation of the microtubules was observed in both sensitive and resistant cells (Figure 6e and f). So, the complete disassembly of the microtubular network occurred at equivalent navelbine concentrations in J82-NVB and in J82 cells. The reversibility of the depolymerisation mechanism was also tested. When the cells were treated with 2,000 nM navelbine for 1 h and then incubated for an additional 6 h in drug-free medium, reassembly of microtubules was observed only in resistant and not in sensitive cells (Figure 6g and h). The same result was obtained with vinblastine. On the contrary, no reversibility was observed after treatment of the cells with 2,000 nM colchicine (not shown).

### *Effect of cycloheximide on cell resistance and on reassembly of microtubules*

We have tested the effect of cycloheximide, an inhibitor of protein synthesis, on the resistance of J82-NVB cells. The growth inhibition assay was performed as described in the

Materials and methods section. J82 and J82-NVB cells were incubated for 1 h in medium containing navelbine with or without 50 $\mu$g ml$^{-1}$ cycloheximide. The cells were then washed and incubated in drug-free medium for 72 h. Cell numbers were then determined. The $IC_{50}$ of navelbine was not modified by the treatment with cycloheximide in sensitive cells but was slightly decreased in resistant cells (Table IV). The resistance factor was decreased from 17 to 11. The cells were also treated for 1 h in medium containing 2,000 nM navelbine and 50 $\mu$g ml$^{-1}$ cycloheximide and then incubated for 6 h in medium containing 50 $\mu$g ml$^{-1}$ cycloheximide but without navelbine. Microtubules were studied as described above. Reassembly of microtubules was observed in resistant cells even in the presence of cycloheximide (data not shown).

**Figure 6** Study of the microtubular network of J82 and J82-NVB cells using immunofluorescence. J82 (**a**, **c**, **e** and **g**) and J82-NVB (**b**, **d**, **f** and **h**) cells were incubated for 1 h in RPMI medium, free of drug (**a** and **b**) or containing 200 nM (**c** and **d**) or 2,000 nM (**e** and **f**) navelbine. In order to study the reversibility of microtubule depolymerisation, the cells, incubated for 1 h with 2,000 nM navelbine, were incubated for an additional 6 h in drug-free medium (**g** and **h**). The cells were fixed and tubulin was stained using a mouse anti-$\alpha$-tubulin antibody followed by a fluorescein-conjugated anti-mouse antibody. The slides were viewed with a laser scanning confocal fluorescence microscope (MRC 600, Bio-Rad). Bar = 15 $\mu$m.

### Discussion

In the present study, we have described the characteristics of a bladder carcinoma cell line, J82-NVB, selected for resistance to navelbine. The results show that the resistance phenotype of this cell line is not a MDR phenotype. P-glycoprotein was not detected in the membrane of J82-NVB cells. Atypical resistance phenotype was confirmed by the lack of cross-resistance to MDR drugs such as doxorubicin, epipodophyllotoxin and colchicine, by the absence of increase in navelbine efflux and by the fact that a reduced accumulation of the drug could not account for the observed resistance level. At equally toxic concentrations, J82-NVB cells accumulate more navelbine than J82 cells. Moreover, verapamil, a drug known to reverse multidrug resistance (Tsuruo *et al.*, 1982; Cass *et al.*, 1989), did not decrease the relative resistance of J82-NVB cells to navelbine compared with sensitive cells.

Few reports have described navelbine resistance mechanisms. We had already shown (Debal *et al.*, 1992) that in the K562-ADM cell line, which is a classic MDR cell line (Tsuruo *et al.*, 1986*b*) selected for resistance to doxorubicin, navelbine accumulation was greatly decreased compared with the sensitive K562 cells. We report here that three K562 resistant sublines selected for resistance in the presence of navelbine are also classic MDR cell lines with increased expression of P-glycoprotein, cross-resistance to navelbine and doxorubicin and reduced drug accumulation. So, it appears that the non-MDR phenotype observed in J82-NVB cells is not due to the use of navelbine as a resistance inducer but is rather a characteristic of this cell line or of the procedure used for the resistance induction. It has been reported that J82 cells selected for resistance to 1 $\mu$M navelbine (100-fold more than for our cells) displayed a classic MDR phenotype (Etievant *et al.*, 1993). Thus, it appears that the resistance phenotype observed is linked to the concentration of drug used for the resistance selection. Such a relationship has already been reported in other cell lines in which non-P-glycoprotein mechanisms of resistance preceded P-glycoprotein expression (Baas *et al.*, 1990).

J82 cells have been described as poorly differentiated epithelial cells with a heterogeneous population morphology (O'Toole *et al.*, 1978). Some morphological differences appear between J82 and J82-NVB cells. The J82 cell population displays both epithelial and fibroblastic morphologies while almost all J82-NVB cells are of the epithelial type. This is accompanied by a greater dispersion of the sensitive cells in non-confluent monolayers. It has been reported that in cells cultured as monolayers cell confluence could play a role in cell resistance. A decreased drug influx in confluent cells compared with non-confluent cells could be responsible for a decreased drug cytotoxicity (Pelletier *et al.*, 1990). We have studied navelbine accumulation and cytotoxicity in confluent and non-confluent J82 and J82-NVB cell monolayers. We have shown that cell confluence could induce some resistance to navelbine in J82 cells and to a lower extent in J82-NVB cells. However, the difference of cohesion between J82 and J82-NVB cell monolayers can only account for a small part of the level of resistance to navelbine, and this resistance level could only be explained by the involvement of some other mechanism.

Apart from mechanism involving a drecreased accumulation of the drug, other types of resistance to vinca alkaloids have been described. These drugs have been reported to affect glutathione metabolism (Beck, 1980; Whelan *et al.*,

**Table IV** Influence of cycloheximide on the growth-inhibitory effect of navelbine

| *Drug* | *$IC_{50}$ (nM)* *J82* | *J82-NVB* | *Resistance factor* |
|---|---|---|---|
| Navelbine | 202 ± 29 | 3,443 ± 614 | 17 |
| Navelbine + cycloheximide 50 $\mu$g ml$^{-1}$ | 259 ± 42 | 2,906 ± 350 | 11 |

1992), but the involvement of this mechanism in vinca alkaloid resistance has not been proved. Intracellular redistribution of the drug should also be considered since such a mechanism has been described in several resistant cell lines (Hindenburg, 1987; Cole, 1992). However, this mechanism could induce resistance to several functionally unrelated drugs while J82-NVB cell resistance is limited to vinca alkaloids and taxoids which share a common target site: tubulin (Gueritte-Voegelein *et al.*, 1991; Jordan *et al.*, 1991). Therefore, J82-NVB cell resistance is most probably linked to the alteration of some microtubule property. Several reports have described various modifications of tubulin in vinca alkaloid-resistant cell lines. A diminished amount (Tsuruo *et al.*, 1986*a*; Ohta *et al.*, 1993) or an increased amount of tubulin has been described in resistant cell lines. However, the study of α- and β-tubulin expression showed no difference between J82 and J82-NVB cell lines. Structural alterations of tubulin in resistant cells could lessen the affinity of tubulin for the drug (Pain *et al.*, 1988) or induce the hyperstabilisation of the microtubules (Cabral & Barlow, 1989). However, cells possessing hyperstable microtubules are cross-resistant to depolymerising drugs such as vinca alkaloids and colchicine and hypersensitive to stabilising drugs such as taxol (Keates *et al.*, 1981; Minotti *et al.*, 1991). This is not the case for our J82-NVB cells, which are sensitive to colchicine and cross-resistant to taxol and taxotere. So, it seems unlikely that hyperpolymerisation of tubulin could be responsible for J82-NVB cell resistance. Moreover, the amounts of polymerised tubulin were found to be similar in J82 and J82-NVB cells.

We have shown that the resistance of J82-NVB cells is linked to the differentiation state of the cells (V. Debal *et al.*, submitted). When the resistant cells were differentiated by retinoic acid, their resistance was lost. This shows that the mechanism of resistance is not due to tubulin mutation, but rather to modifications of some mechanism regulating microtubule dynamics.

Using immunofluorescence, we have studied the microtubules of J82 and J82-NVB cells treated with navelbine. After incubation with navelbine, depolymerisation of the microtubules occurred at nearly the same drug concentration in sensitive and in resistant cells. This shows that even in resistant cells the drug reached its target and was not sequestered in some subcellular compartment. Microtubule depolymerisation in J82 cells treated for 1 h with navelbine occurred approximately at the drug concentration which induced a growth inhibition, while in J82-NVB cells growth inhibition occurred at concentrations higher than those inducing depolymerisation. So, the effect of the drug on microtubules was nearly the same in sensitive and in resistant cells, but resistant cells could survive after removal of the drug whereas sensitive cells could not. Thus, recovery from the inhibition of microtubule dynamics induced by navelbine is probably responsible for the resistance of J82-NVB cells.

In order to study this recovery mechanism, we have tested the reversibility of microtuble depolymerisation in sensitive and resistant cells. After treatment with 2,000 nM navelbine, reassembly of the microtubular network was observed in resistant but not in sensitive cells. The same result was obtained with vinblastine. Moreover, no reversibility was observed after treatment with colchicine, to which the cells are not resistant. This confirms that the mechanism of resistance of J82-NVB cells is at the level of microtubules and involves recovery of microtubule dynamics.

Some mechanisms allowing recovery from cellular damage induced by protein alteration have already been described. They generally involve the synthesis of proteins of the family of molecular chaperones (Ellis & Van der Vries, 1991). Moreover, molecular chaperone proteins play an essential role in the *in vivo* assembly of microtubules (Gupta, 1990). Some of these proteins have been reported to induce cell resistance to vinca alkaloids (Huot *et al.*, 1991; Lee *et al.*, 1992), and these newly synthesised proteins have been found to bind to tubulin in these resistant cells (Lee *et al.*, 1992). We have tested the effect of cycloheximide, an inhibitor of protein synthesis, on the resistance of J82-NVB cells to navelbine. The presence of cycloheximide during the incubation of J82-NVB cells with navelbine results in only a slight decrease of resistance, and reassembly of microtubules occurs in J82-NVB cells even in the presence of cycloheximide. Thus it seems that protein synthesis is not essential for the recovery to occur.

The cross-resistance observed between vinca alkaloids and taxoids may appear rather surprising since vinca alkaloids, like colchicine, are microtubule-depolymerising drugs, contrary to taxol, which promotes tubulin polymerisation (Schiff *et al.*, 1979). However, it has been shown that the antiproliferative action of vinca alkaloids at low concentration results from stabilisation of microtubule dynamic instability rather than from depolymerisation of microtubules (Jordan *et al.*, 1991; Toso *et al.*, 1993). Recently, it has been shown that taxol, at low concentration, shares a common antiproliferative mechanism with vinca alkaloids (Jordan *et al.*, 1993). In our cells, low concentrations of the drug remained after removal of the drug from the medium. Under these conditions, the mechanism of action of vinca alkaloids and taxol analogues are similar. Moreover, cross-resistance between vinca alkaloids and taxol has already been observed in another non-MDR cell line resistant to microtubule poisons (Ohta *et al.*, 1993). Other authors have studied the reversibility of microtubules bundling induced by taxol in different leukaemia cell lines (Rowinsky *et al.*, 1988). They concluded that in cells that were relatively resistant to taxol microtubule bundling was reversible, unlike in relatively sensitive cells, in which microtubule bundles persisted.

All these results are consistent with our study, which suggests that the mechanism of resistance of J82-NVB cells is mainly recovery from the inhibition of microtubule dynamism induced by the drug.

This work was supported by Laboratoires Pierre Fabre Médicament (France). We thank Dominque Ploton (INSERM U314) for his technical assistance with the confocal microscope, Gérard Simon for performing cytometric analysis of P-glycoprotein, Yves Carpentier for cell cycle analysis, Jean-François Riou for Northern blot analysis, Daniel Leynadier for help with microtubule immunostaining and Ganesh D. Sockalingum for his helpful English revision of our manuscript.

### References

BAAS, F., JONGSMA, A.P.M., BROXTERMAN, H.J., ARCECI, R.J., HOUSMAN, A.D., SCHEFFER, G.L., RIETHORST, A., VAN GROENIGEN, M., NIEUWINT, A.W.M. & JOENJE, H. (1990). Non-P-glycoprotein mediated mechanism for multidrug resistance precedes P-glycoprotein expression during *in vitro* selection for doxorubicin resistance in a human lung cancer cell line. *Cancer Res.*, **50**, 5392–5398.

BECK, W.T. (1980). Increase by vinblastine of oxidized glutathione in cultured mammalian cells. *Biochem. Pharmacol.*, **29**, 2333–2337.

BECK, W.T. (1987). The cell biology of multiple drug resistance. *Biochem. Pharmacol.*, **36**, 2879–2887.

BINET, S., FELLOUS, A., KRIKORIAN, A., COUZINIER, J.P. & MEININGER, V. (1989). In situ analysis of the action of navelbine on various types of microtubules using immunofluorescence. *Semin. Oncol.*, **16**, 5–8.

CABRAL, F. & BARLOW, S. (1989). Mechanisms by which mammalian cells acquire resistance to drugs that affect microtubule assembly. *FASEB J.*, **3**, 1593–1599.

CASS, C.E., JANOWSKA-WIECZOREK, A., LYNCH, M.A., SHEININ, H., HINDENBURG, A.A. & BECK, W.T. (1989). Effect of duration of exposure to verapamil on vincristine activity against multidrug-resistant human leukemic cell lines. *Cancer Res.*, **49**, 5798–5804.

COLE, S.P.C., BHARWAJ, G., GERLACH, J.H., MACKIE, J.E., GRANT, C., ALMQUIST, K.C., STEWART, A.J., KURZ, E.U., DUNCAN, A.M.V. & DEELEY, R.G. (1992). Overexpression of a transporter gene in a multidrug-resistant human lung cancer cell line. *Science*, **258**, 1650–1654.

CORDELL, J.L., FALINI, B., ERBER, W.N., GHOSH, A.K., ABDULAZIZ, Z., MACDONALD, S., PULFORD, K.A., STEIN, H. & MASON, D.Y. (1984). Immunoenzymatic labeling of monoclonal antibodies using immune complexes of alkaline phosphatase and monoclonal anti-alkaline phosphatase (APAAP complexes). *J. Histochem. Cytochem.*, **32**, 219–229.

COWAN, N.J., DOBNER, P.R., FUCHS, E.V. & CLEVELAND, D.W. (1983). Expression of human α-tubulin genes: interspecies conservation of 3′ untranslated regions. *Mol. Cell. Biol.*, **3**, 1738–1745.

CUVIER, C., ROBLOT-TREUPEL, L., MILLOT, J.M., LIZARD, G., CHEVILLARD, S., MANFAIT, M. & POUPON, M.F. (1992). Doxorubicin-loaded nanospheres bypass tumor cell multidrug resistance. *Biochem. Pharmacol.*, **44**, 509–517.

DEBAL, V., MORJANI, H., MILLOT, J.M., ANGIBOUST, J.F., GOURDIER, B. & MANFAIT, M. (1992). Determination of vinorelbine (navelbine) in tumour cells by high-performance liquid chromatography. *J. Chromatogr.*, **581**, 93–99.

DE INES, C., LEYNADIER, D., BARASOAIN, I., PEYROT, V., GARCIA, P., BRIAND, C., RENER, G.A. & TEMPLE, C. (1994). Inhibition of microtubules and cell cycle arrest by a new 1-deaza-7,8-dihydropteridine antitumor drug, CI 980, and by its chiral isomer, NSC 613863. *Cancer Res.*, **54**, 75-84.

ELLIS, R.J. & VAN DER VIES, S.M. (1991). Molecular chaperones. *Annu. Rev. Biochem.*, **60**, 321–347.

ENDICOTT, J.A. & LING, V. (1989). The biochemistry of P-glycoprotein-mediated multidrug resistance. *Annu. Rev. Biochem.*, **58**, 137–171.

ETIEVANT, C., PAUWELS, O. & KISS, R. (1993). Digital cell image analysis of verapamil-induced effects in chemosensitive and chemoresistant neoplastic cell lines. *J. Cancer Res. Clin. Oncol.*, **120**, 76–84.

FISZER-SZAFARZ, B., SZAFARZ, D. & DE MURILLO, AG. (1981). A general, fast, and sensitive micromethod for DNA determination: application to rat and mouse liver, rat hepatoma, human leukocyte, chicken fibroblasts and yeast cells. *Anal. Biochem.*, **110**, 165–170.

GIGLI, M., DOGLIA, S.M., MILLOT, J.M., VALENTINI, L. & MANFAIT, M. (1988). Quantitative study of doxorubicin in living cell nuclei by microspectrofluorometry. *Biochim. Biophys. Acta*, **950**, 13–20.

GIGLI, M., ROSOANAIVO, T.D.W., MILLOT, J.M., JEANNESSON, P., RIZZO, V., JARDILLIER, J.C., ARCAMONE, F. & MANFAIT, M. (1989). Correlation between growth inhibition and intranuclear doxorubicin and 4′-deoxy-4′-iododoxorubicin quantitated in living K562 cells by microspectrofluorometry. *Cancer Res.*, **49**, 560–564.

GUERITTE-VOEGELEIN, F., GUENARD, D., LAVELLE, F., LE GOFF, M.T., MANGATAL, L. & POTIER, P. (1991). Relationships between the structure of taxol analogues and their antimitotic activity. *J. Med. Chem.*, **34**, 992–998.

GUPTA, R.S. (1990). Mitochondria, molecular chaperone proteins and the in vivo assembly of microtubules. *Trends Biochem. Sci.*, **15**, 415–418.

HABER, M., NORRIS, M.D., KAVALLARIS, M., BELL, D.R., DAVEY, R.A., WHITE, L. & STEWART, B. (1989). Atypical multidrug resistance in a therapy-induced drug resistant human leukemia cell line (LALW-2): resistance to vinca alkaloids independent of P-glycoprotein. *Cancer Res.*, **49**, 5281–5287.

HALL, J.L., DUDLEY, L., DOBNER, P.R., LEWIS, S.A. & COWAN, N.J. (1983). Identification of the two human β-tubulin isotypes. *Mol. Cell. Biol.*, **3**, 854–862.

HINDENBURG, A.A., BAKER, M.A., GLEYZER, E., STEWART, V.J., CASE, N. & TAUB, R.N. (1987). Effect of verapamil and other agents on the distribution of anthracyclines and on reversal of drug resistance. *Cancer Res.*, **47**, 1421–1425.

HOUGHTON, J.A., HOUGHTON, P.J., HAZELTON, B.J. & DOUGLAS, E.D. (1985). In situ selection of a human rhabdomyosarcoma resistant to vincristine with altered β-tubulins. *Cancer Res.*, **45**, 2706–2712.

HUOT, J., ROY, G., LAMBERT, H., CHRETIEN, P. & LANDRY, J. (1991). Increased survival after treatments with anticancer agents of Chinese hamster cells expressing the human $M_r$ 27000 heat shock protein. *Cancer Res.*, **51**, 5245–5252.

JORDAN, M.A., THROWER, D. & WILSON, L. (1991). Mechanism of inhibition of cell proliferation by vinca alkaloids. *Cancer Res.*, **51**, 2212–2222.

JORDAN, M.A., TOSO, R.J., THROWER, D. & WILSON, L. (1993). Mechanism of mitotic block and inhibition of cell proliferation by taxol at low concentrations. *Proc. Natl Acad. Sci. USA*, **90**, 9552–9556.

KARTNER, N., RIORDAN, J.R. & LING, V. (1983). Cell surface P-glycoprotein associated with multidrug resistance in mammalian cell lines. *Science*, **222**, 1285–1288.

KEATES, R.A.B., SARANGI, F. & LING, V. (1981). Structural and functional alterations in microtubule protein from Chinese hamster ovary cell mutant. *Proc. Natl Acad. Sci. USA*, **78**, 5638–5642.

LEE, W.C., LIN, K.Y., CHEN, K.D. & LAI, Y.K. (1992). Induction of Hsp 70 is associated with vincristine resistance in heat-shocked 9L rat brain tumour cells. *Br. J. Cancer*, **66**, 653–659.

LOZZIO, C.B. & LOZZIO, B.B. (1975). Human chronic myelogenous leukemia cell line with positive philadelphia chromosome. *Blood*, **45**, 321–334.

MCCLEAN, S. & HILL, B.T. (1992). An overview of membrane, cytosolic and nuclear proteins associated with the expression of resistance to multiple drugs in vitro. *Biochim, Biophys. Acta*, **1114**, 107–127.

MINOTTI, A.M., BARLOW, S.B. & CABRAL, F. (1991). Resistance to antimitotic drugs in Chinese hamster ovary cells correlates with changes in the level of polymerized tubulin. *J. Biol. Chem.*, **266**, 3987–3994.

OHTA, S., NISHIO, K., KUBO, S., NISHIO, M., OHMORI, T., TAKAHASHI, T. & SAIJO, N. (1993). Characterisation of a vindesine-resistant human small-cell lung cancer cell line. *Br. J. Cancer*, **68**, 74–79.

O'TOOLE, C., PRICE, Z.H., OHNUKI, Y. & UNSGAARD, B. (1978). Ultrastructure, karyology and immunology of a cell line originated from a human transitional-cell carcinoma. *Br. J. Cancer*, **38**, 64–76.

PAIN, J., SIROTNAK, F.M., BARRVECO, J.R., YANG, C.H. & BIEDLER, J.L. (1988). Altered molecular properties of tubulin in a multidrug resistant variant of chinese hamster cells selected for resistance to vinca alkaloids. *J. Cell Physiol.*, **136**, 341–347.

PASTAN, I. & GOTTESMAN, M. (1987). Multiple drug resistance in human cancer. *N. Engl. J. Med.*, **316**, 1388–1397.

PAUWELS, O. & KISS, R. (1991). Digital morphonuclear analyses of sensitive versus resitant neoplastic cells to vinca alkaloid, alkylating, and intercalating drugs. *Cytometry*, **12**, 388–397.

PELLETIER, H., MILLOT, J.M., CHAUFFERT, B., MANFAIT, M., GENNE, P. & MARTIN, F. (1990). Mechanisms of resistance of confluent human and rat colon cancer cells to anthracyclines: alteration of drug passive diffusion. *Cancer Res.*, **50**, 6626–6631.

POTIER, P. (1989). The synthesis of navelbine prototype of a new series of vinblastine derivatives. *Semin. Oncol.*, **16**, 2–4.

ROWINSKY, E.K., DONEHOWER, R.C., JONES, R.J. & TUCKER, R.W. (1988). Microtubule changes and cytotoxicity in leukemic cell lines treated with taxol. *Cancer Res.*, **48**, 4093–4100.

SAMBROOK, J., FRITSCH, E.F. & MANIATIS, T. (1989). *Molecular Cloning, a Laboratory Manual*, 2nd edn. Cold Spring Harbor Laboratory Press: Cold Spring Harbor, NY.

SCHIFF, P.B., FANT, J. & HORWITZ, S.B. (1979). Promotion of microtubule assembly in vitro by taxol. *Nature*, **277**, 665–667.

TOSO, R.J., JORDAN, M.A., FARREL, K.W., MATSUMOTO, B. & WILSON, L. (1993). Kinetic stabilization of microtubule dynamic instability in vitro by vinblastine. *Biochemistry*, **32**, 1285–1293.

TSURUO, T., IIDA, H., TSUKAGOSHI, S. & SAKURAI, Y. (1982). Increased accumulation of vincristine and adriamycin in drug-resistant P388 tumor cells following incubation with calcium antagonists and calmodulin inhibitors. *Cancer Res.*, **42**, 4730–4733.

TSURUO, T., OH-HARA, T. & SAITO, H. (1986*a*). Characteristics of vincristine resistance in vincristine resistant human myelogenous leukemia k562. *Anticancer Res.*, **6**, 637–642.

TSURUO, T., IIDA, H., KAWABATA, H., OH-HARA, T., HAMADA, H. & UTAKOJI, T. (1986*b*). Characteristics of resistance to adriamycin in human myelogenous leukemia K562 resistant to adriamycin and in isolated clones. *Jpn J. Cancer Res.*, **77**, 682–692.

VINDELOV, L.L., CHRISTENSEN, I.J. & NISSEN, N.I. (1983). A detergent trypsin method for the preparation of nuclei for flow cytometric DNA analysis. *Cytometry*, **3**, 321–327.

WHELAN, R.D.H., WARING, C.J., WOLF, C.R., HAYES, J.D., HOSKING, L.K. & HILL, B.T. (1992). Over-expression of P-glycoprotein and glutathione S-transferase PI in MCF-7 cells selected for vincristine resistance in vitro. *Int. J. Cancer*, **52**, 241–246.